# A CYP21A2 based whole-cell system in *Escherichia coli* for the biotechnological production of premedrol

**DOI:** 10.1186/s12934-015-0333-2

**Published:** 2015-09-15

**Authors:** Simone Brixius-Anderko, Lina Schiffer, Frank Hannemann, Bernd Janocha, Rita Bernhardt

**Affiliations:** Department of Biochemistry, Saarland University, 66123 Saarbrücken, Germany; Sanofi-Aventis Deutschland GmbH, C&BD Frankfurt Biotechnology, 65926 Frankfurt-Höchst, Germany

**Keywords:** Methylprednisolone, Medrane, CYP21A2, Cytochrome P450, Whole-cell biocatalysis, *E. coli*, Etp1^fd^, Arh1, CPR, Adx, Steroid

## Abstract

**Background:**

Synthetic glucocorticoids like methylprednisolone (medrol) are of high pharmaceutical interest and represent powerful drugs due to their anti-inflammatory and immunosuppressive effects. Since the chemical hydroxylation of carbon atom 21, a crucial step in the synthesis of the medrol precursor premedrol, exhibits a low overall yield because of a poor stereo- and regioselectivity, there is high interest in a more sustainable and efficient biocatalytic process. One promising candidate is the mammalian cytochrome P450 CYP21A2 which is involved in steroid hormone biosynthesis and performs a selective oxyfunctionalization of C21 to provide the precursors of aldosterone, the main mineralocorticoid, and cortisol, the most important glucocorticoid. In this work, we demonstrate the high potential of CYP21A2 for a biotechnological production of premedrol, an important precursor of medrol.

**Results:**

We successfully developed a CYP21A2-based whole-cell system in *Escherichia coli* by coexpressing the cDNAs of bovine *CYP21A2* and its redox partner, the NADPH-dependent cytochrome P450 reductase (*CPR*), via a bicistronic vector. The synthetic substrate medrane was selectively 21-hydroxylated to premedrol with a max. yield of 90 mg L^−1^ d^−1^. To further improve the biocatalytic activity of the system by a more effective electron supply, we exchanged the CPR with constructs containing five alternative redox systems. A comparison of the constructs revealed that the redox system with the highest endpoint yield converted 70 % of the substrate within the first 2 h showing a doubled initial reaction rate compared with the other constructs. Using the best system we could increase the overall yield of premedrol to a maximum of 320 mg L^−1^ d^−1^ in shaking flasks. Optimization of the biotransformation in a bioreactor could further improve the premedrol gain to a maximum of 0.65 g L^−1^ d^−1^.

**Conclusions:**

We successfully established a CYP21-based whole-cell system for the biotechnological production of premedrol, a pharmaceutically relevant glucocorticoid, in *E. coli* and could improve the system by optimizing the redox system concerning reaction velocity and endpoint yield. This is the first step for a sustainable replacement of a complicated chemical low-yield hydroxylation by a biocatalytic cytochrome P450-based whole-cell system.

**Electronic supplementary material:**

The online version of this article (doi:10.1186/s12934-015-0333-2) contains supplementary material, which is available to authorized users.

## Background

Since the 1950’s, the development of synthetic glucocorticoids is of growing interest with the aim to substitute the natural steroid hormone hydrocortisone as therapeutical compound. The superficial aim is to reduce severe hydrocortisone induced side effects, such as the disturbance of the electrolyte homeostasis, and to synthesize molecules with increased anti-inflammatory effects [1]. Based upon the artificial hydrocortisone derivative prednisolone the highly effective compound medrol (6-methylprednisolone) was developed by addition of a methyl group at carbon atom 6. Medrol turned out to have a far higher glucocorticoid activity than hydrocortisone without a comparative increase of electrolyte activity [2]. Medrol was synthesized from its precursor premedrol via a simple to perform biotechnological 1,2 dehydrogenation [3]. Today, medrol is a widespread drug in the treatment of autoimmune diseases, allergic reactions, multiple sclerosis and rheumatic arthritis [4]. Therefore, the demand for this pharmaceutically highly relevant glucocorticoid is still increasing. One bottleneck during contemporary premedrol and, therefore, medrol production is the hydroxylation of carbon atom 21. The chemical introduction of a hydroxyl group into a steran scaffold consists of a multistep-synthesis with the necessity to apply protecting groups and toxic compounds like iodine [5]. As by-products occur after each reaction step, a time consuming chromatographic purification is necessary, which leads to a reduced overall yield and a low efficiency factor [6, 7]. With regard to a more sustainable and less polluting production process and a regio- and stereoselective oxyfunctionalization at C21, the focus has shifted from the chemical process to an enzyme based biotechnological production of medrol and its precursor. A promising candidate for the enzymatic reaction is the mammalian cytochrome P450 21-hydroxylase (CYP21A2), which is a member of the cytochrome P450 (CYP, P450) superfamily. CYP21A2 is a protein of the endoplasmic reticulum and plays a crucial role in steroid hormone biosynthesis by providing the precursors of the most important mineralocorticoid, aldosterone, and the main glucocorticoid, cortisol, via a highly selective 21-hydroxylation, which is ensured by a unique amino acid arrangement within the active site [8–10]. A sufficient electron supply for the hydroxylation reaction is realized by its natural redox partner, the NADPH-dependent cytochrome P450 reductase (CPR), a membrane bound protein as well [11, 12]. Cytochromes P450 are external monooxygenases and exhibit, when reduced and in complex with CO, a unique absorption maximum at 450 nm due to the cysteinate coordinated heme group at the active site [13]. Their ability to functionalize molecular oxygen empowers them to catalyze a broad range of reactions, such as hydroxylations and even a chemically difficult to perform C–C bond cleavage. Apart from steroid hormone biosynthesis, they act as main detoxifying enzymes in the liver and are, therefore, involved in xenobiotics and drug metabolism. P450s are able to convert a great variety of substrates like steroids, terpenes as well as fatty acids, which shows their high potential as versatile biocatalysts [14, 15]. Since the 1960s, cytochromes P450 are crucially involved in the glucocorticoid synthesis in large scale by fermentation of species of the fungus *Curvularia,* whose later characterized P450 system was shown to be able to convert 11-deoxycortisol to cortisol [16–18]. In 2003, the application of a modified *Saccharomyces cerevisiae* strain was published, which performs cortisol production from a simple carbon source [19]. Aside from genetic manipulation of the yeast’s ergosterol synthesis pathway, CYPs involved in steroid hormone synthesis, including CYP21A2, were expressed in this yeast strain, which shows the high importance of these enzymes for stereo- and regioselective steroid hydroxylation. To date, CYPs find their application in various industrial production processes and the number is still growing. Efforts are made to design whole-cell systems with single CYPs for the desired reaction in a suitable host to avoid by-products originating from homologous CYP systems like in case of *C. lunata*. Previously, a heterologous human CYP11B1 whole-cell system for a more selective cortisol production has been published [20]. Biocatalysis with whole cells ensures a better protein stability and a supply with costly cofactors, such as NADPH [21]. Concerning mammalian CYP21A2, a whole-cell system in the yeast *Schizosaccharomyces pombe* was already established with human CYP21A2, but with limited success due to a low recombinant protein yield and the host’s long lasting generation time [22]. In other approaches to develop CYP based whole-cell systems, *Escherichia coli* emerged to be a suitable host attributed to its short generation time and the lack of intrinsic CYP systems [23]. Functional bovine CYP21A2 could already be successfully expressed with high yield in *E. coli* [24, 25]. These fundamentals were the starting point for our efforts to establish an efficient CYP21A2-based whole-cell system in *E. coli* for the production of premedrol, the precursor of medrol, via a simple one-step hydroxylation at C21. In the following, we demonstrate the successful expression and purification of bovine CYP21A2 and in vitro studies concerning the substrate-protein-interaction, the development of a biotransformation in whole cells and an improvement of the biocatalytic efficiency by using alternative redox systems for a more sufficient electron supply.

## Results and discussion

### Protein purification and in vitro characterization

#### Purification of bovine CYP21A2

**Fig. 1:**
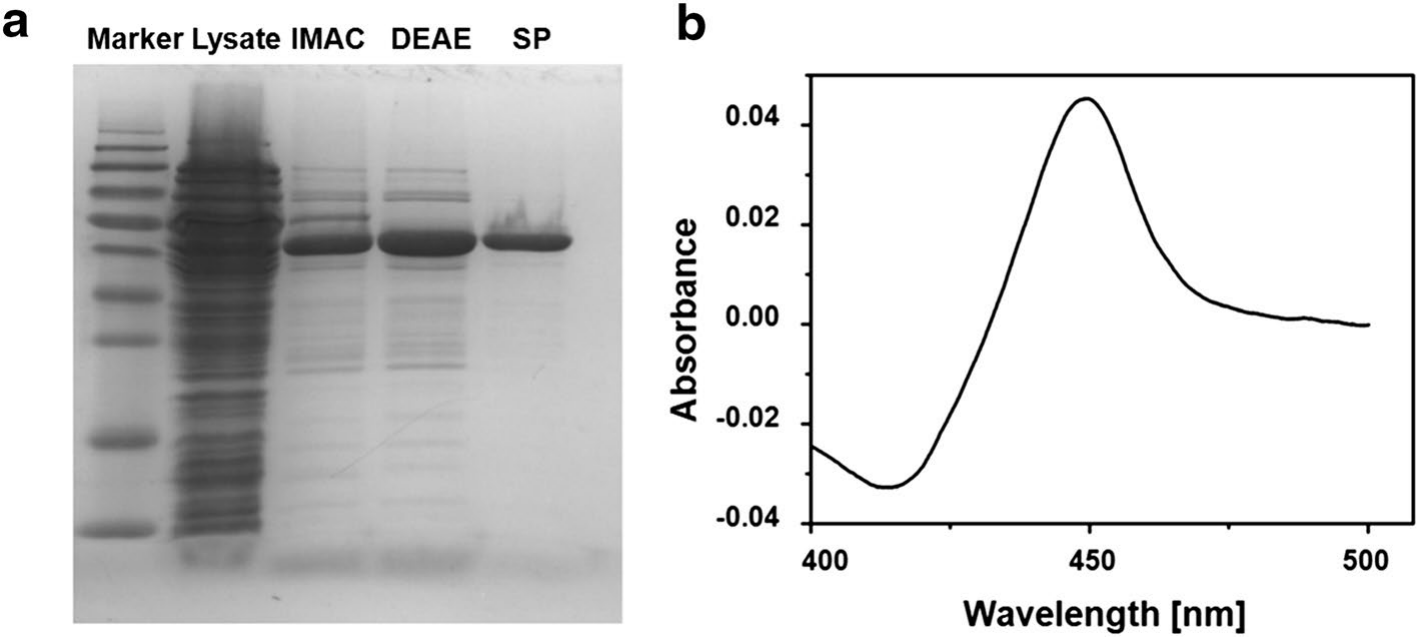
Protein analysis of purified bovine CYP21A2 after three chromatographic steps. **a** SDS-PAGE analysis and protein staining shows the purification steps of CYP21A2 via IMAC, DEAE- and SP-Sepharose resulting in a single protein band with a size of approx. 55 kDa (estimated weight: 54.6 kDa); **b** Difference spectroscopy of purified bovine CYP21A2 was performed showing a typical absorption maximum at 450 nm in the reduced state in complex with CO

Since bovine CYP21A2 could already be expressed in *E. coli*, we chose this mammalian CYP21A2 isoform for the initial examination of its suitability for a whole-cell system in *E. coli.* To ascertain whether bovine CYP21A2 is able to convert medrane to premedrol by a stereoselective 21-hydroxylation, the protein had to be expressed and purified according to Arase et al. [24]. The cDNA was subcloned into a pET17b vector, resulting in the vector pET17b_21b. The vector was co-transformed with the vector pGro12, which encodes for the *E. coli* chaperones GroEL/ES, into C43(DE3) cells. After protein expression, cell lysis took place via sonification for the subsequent purification. The purification was performed via IMAC, anion and cation exchange chromatography and the protein was analyzed by SDS-PAGE, confirming the estimated molecular weight of 54.6 kDa, and by CO difference spectroscopy, which confirmed a correct insertion of the heme prosthetic group by showing a typical absorption maximum at 450 nm without a hint of inactive protein, indicated by a peak at 420 nm (Fig. 1). Taken together, the expression as well as the purification of bovine CYP21A2 was successful with an expression level of max. 398 nmol L^−1^ culture. The purified enzyme was used for further investigations.

#### In vitro conversion of medrane with purified CYP21A2

**Fig. 2:**
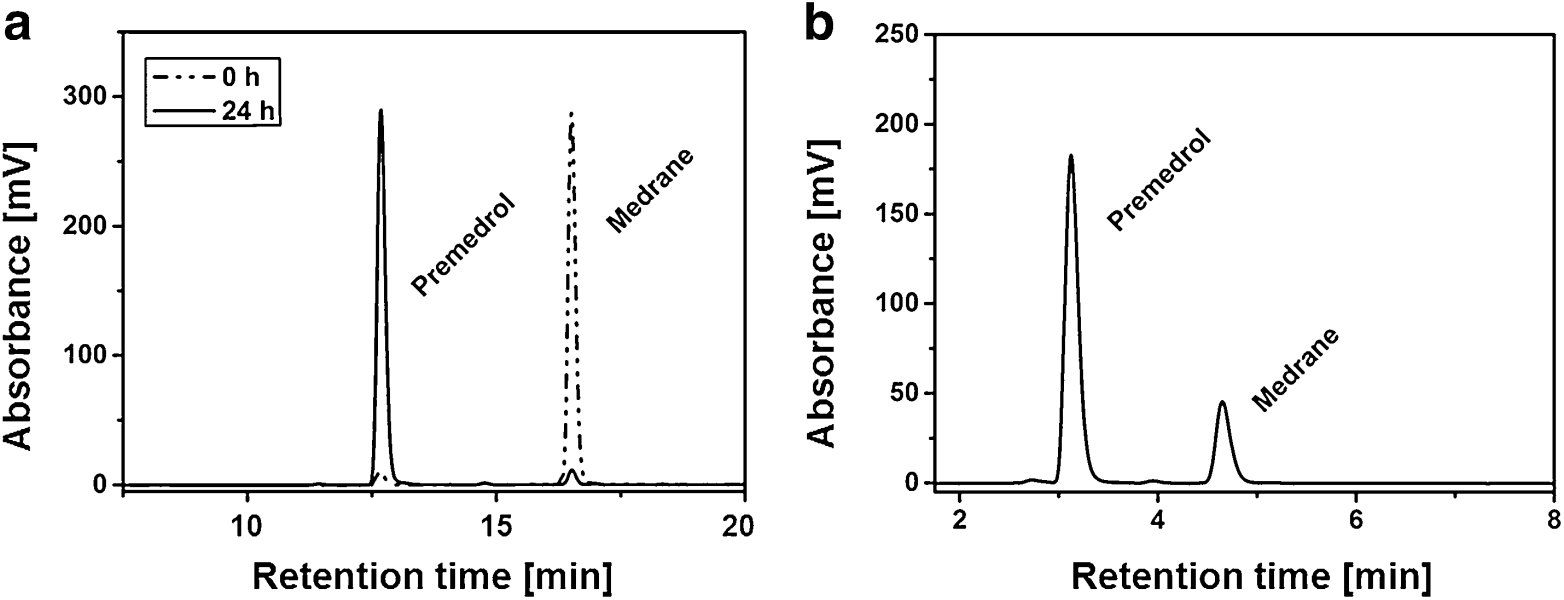
HPLC chromatograms of the in vivo and in vitro conversion of medrane with bovine CYP21A2 and CPR. **a** Medrane was converted with resting cells of C43(DE3) containing the bovine CYP21A2 isoform and CPR encoding vector p21b_bRED. Samples were taken after 24 h and extracted for HPLC analysis. The steroids were separated by an acetonitril:water gradient. **b** Substrate conversions of medrane were performed with purified bovine CYP21A2 and its redox partner CPR for 30 min. Steroids were extracted and analyzed by HPLC to verify a selective conversion of medrane to premedrol. The steroids were separated by an acetonitril:water gradient, showing the 21-hydroxylated product premedrol

In order to prove a selective conversion of medrane to premedrol by a 21-hydroxylation, in vitro assays were carried out to perform a proof-of-principle, as medrane exhibits slight modifications within the steran skeleton compared with the natural CYP21A2 substrates progesterone (P4) and 17OH-progesterone (17OH-P4). Hence, substrate conversion with purified bovine CYP21A2 was performed with the synthetic substrate medrane characterized by its additional methyl group at carbon atom 6 and a hydroxyl group at carbon atom 11 compared with the natural substrate 17OH-P4. HPLC analysis revealed a 21-hydroxylation of medrane and demonstrated a stereoselective production of the wished product premedrol in an efficient biocatalytic one-step hydroxylation without by-product formation (Fig. 2b). It has been shown that bovine CYP21A2 is able to hydroxylate a synthetic substrate of high pharmaceutical interest.

### Development of a whole-cell system for a biocatalytic premedrol production

**Fig. 3:**
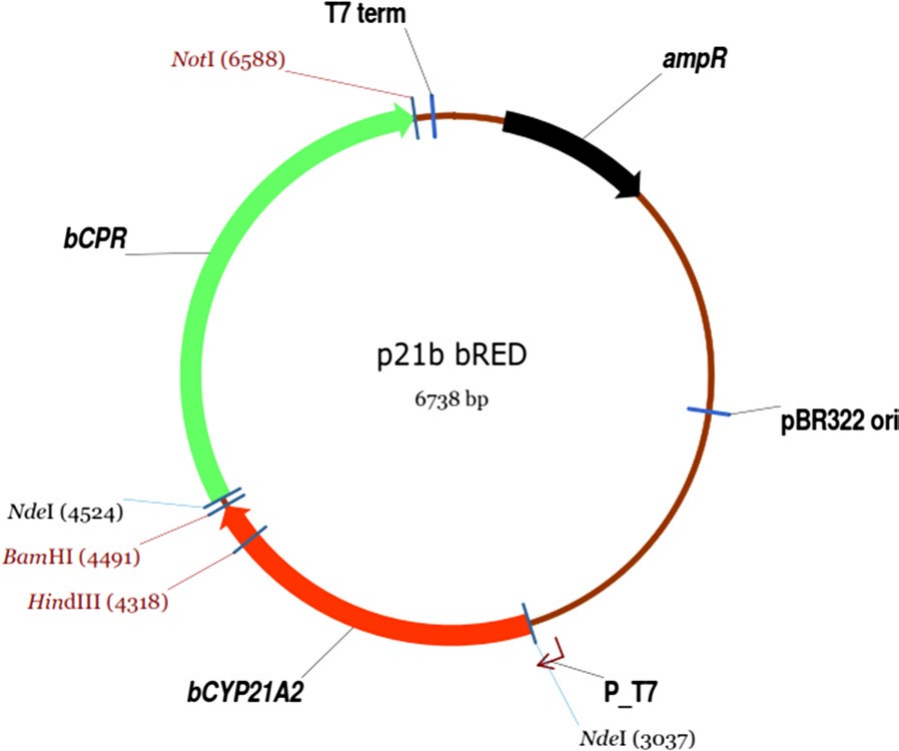
Vector map of p21b_bRED containing the bicistronic transcription unit for bovine CYP21A2 and the CPR. For the whole-cell biotransformation, this bicistronic vector was constructed, based on the pET17b vector with a T7 promoter and an ampicillin resistance gene. The *CPR* cDNA encodes for the full length protein

After a conversion of medrane to premedrol by purified CYP21A2 was verified in vitro, the subsequent experiments focused on an establishment of a biotransformation using whole cells, showing advantages like an improved enzyme stability and the supply of costly co-factors by the cell itself [21]. For the development of a whole-cell system, the bicistronic vector p21b_bRED was constructed, carrying the cDNAs for bovine *CYP21A2* and *CPR* (Fig. 3). The natural redox partner CPR is responsible for electron supply in the endoplasmic reticulum through protein interaction. Cells were co-transformed with the respective vector and with the plasmid pGro12, encoding the chaperones GroEL/ES to ensure a proper folding of the membrane proteins CYP21A2 and CPR [26]. Although complex medium is suitable for bacterial cell growth and supports a high expression yield of recombinant proteins, it is inappropriate for whole-cell biotransformations with cytochromes P450 due to inhibitory effects of medium compounds and *E. coli* metabolites such as indole [27, 28]. For this reason, biotransformation with whole cells was performed with resting cells using potassium phosphate buffer as a conversion medium. As the metabolism, including protein biosynthesis, of resting cells is reduced to a minimum, more co-factors like NADPH can be recruited for the CYP dependent reaction [29]. By the addition of glycerol to the reaction mix, an NADPH-regeneration is ensured by the activity of metabolic enzymes like the isocitrate dehydrogenase. For initial examination, medrane was added to the whole-cell system and samples were taken after 24 h for HPLC analysis. Medrane was converted to premedrol by the constructed system without by-product formation, verifying the highly specific 21-hydroxylation not only with purified enzymes but also by a biotransformation with whole cells (Fig. 2a). With a substrate concentration of 250 µM, a maximum premedrol yield of 93 mg L^−1^ d^−1^ could be achieved. Since the human CYP21A2 isoform shares a 79 % sequence homology to the bovine one, we additionally tested the human CYP21A2 isoform concerning its ability to produce premedrol. Just as the bovine enzyme, it performs a selective 21-hydroxylation of medrane, but exhibits a poor yield with 40 mg L^−1^ d^−1^ (Additional file 1: Fig. S1). Therefore, we concentrated on the bovine isoform in further experiments to improve the whole-cell system’s efficiency.

### Improvement of the whole-cell system by using different redox partners

CYP involved whole-cell systems require an efficient cytochrome P450/redox partner interaction to ensure a sufficient electron supply for the hydroxylation reaction [15]. Therefore, a stabilized redox partner synthesis as well as an optimal interaction with the CYP should be intended. In the following, we focused on these aspects by testing various redox systems of different origin.

#### Selection of alternative redox partners for CYP21

Bovine membrane-bound microsomal CPR represents the naturally occurring redox partner for CYP21 in the endoplasmic reticulum and was therefore the first choice for a co-expression in a whole-cell system. Though, this protein is difficult to produce recombinantly due to its property as a membrane protein. Therefore, efforts were undertaken to search for alternative redox partners of the bovine CYP21A2, which are easily expressed in *E. coli* in sufficient amounts, more stable and solvent resistant. On this account, we concentrated on the participating proteins of three naturally occurring redox systems, each consisting of two components, a ferredoxin or flavodoxin reductase and a ferredoxin as final electron donor for CYP21A2.

First, we focused on the soluble ferredoxin adrenodoxin (Adx), which reconstitutes the mitochondrial electron transfer system together with the membrane associated adrenodoxin reductase (AdR), which receives electrons from NADPH [12, 30–32]. This system is responsible for the electron supply of the mitochondrial CYPs, CYP11A1, CYP11B1 and CYP11B2, which are also involved in steroid hormone biosynthesis. Pechurskaya et al. showed, that Adx is able to transfer electrons to purified CYP21A2, in the case of truncated CYP21A2 even more effectively than the CPR in in vitro assays [33]. In this work, we used the Adx version, Adx_1–108_, which exhibits an increased electron transfer efficiency to some CYPs [34, 35].

Second, a redox system originating from the fission yeast *S. pombe* and consisting of the adrenodoxin reductase homologue 1 (arh1) and the ferredoxin domain of electron transfer protein 1 (etp1^fd^) was considered, since Ewen et al. showed that this system is able to substitute Adx and AdR regarding an electron transfer to CYP11A1 [36]. In *S. pombe,* arh1 and etp1^fd^ are involved in heme biosynthesis in the mitochondrium [37]. Etp1^fd^ as iron-sulfur protein is highly homologous to adrenodoxin and is able to transfer electrons to mammalian steroidogenic CYPs [38, 39]. Here, we used the truncated version of etp1^fd^ (516–618). Both proteins can be produced as cytosolic proteins in *E. coli*. Furthermore, it has been demonstrated, that arh1 can be reduced not only by NADPH like AdR, but also by NADH and that arh1 of *S. cerevisiae* is able to interact with bovine Adx [40]. Regarding a whole-cell system, a second electron pool could be of great advantage for a more efficient hydroxylation rate [36]. Janocha et al. already demonstrated a biotechnological application of arh1 and etp1^fd^ from *S. pombe* with CYP105A1 from *Streptomyces griseolus* [41]. As in previous works of our laboratory, we used an arh1 variant with an improved FAD-binding behavior, ensuring co-factor stability [36].

Third, we applied the *E. coli* NADPH-flavodoxin reductase Fpr as an alternative reductase for a whole-cell system. It has been demonstrated previously that the soluble Fpr is able to transfer electrons to Adx and, therefore, represents an efficient substitution for AdR [27]. The Fpr together with flavodoxin A (FldA) is part of an *E. coli* redox system, which is involved in biosynthetic processes such as amino acid synthesis [42, 43].

**Fig. 4:**
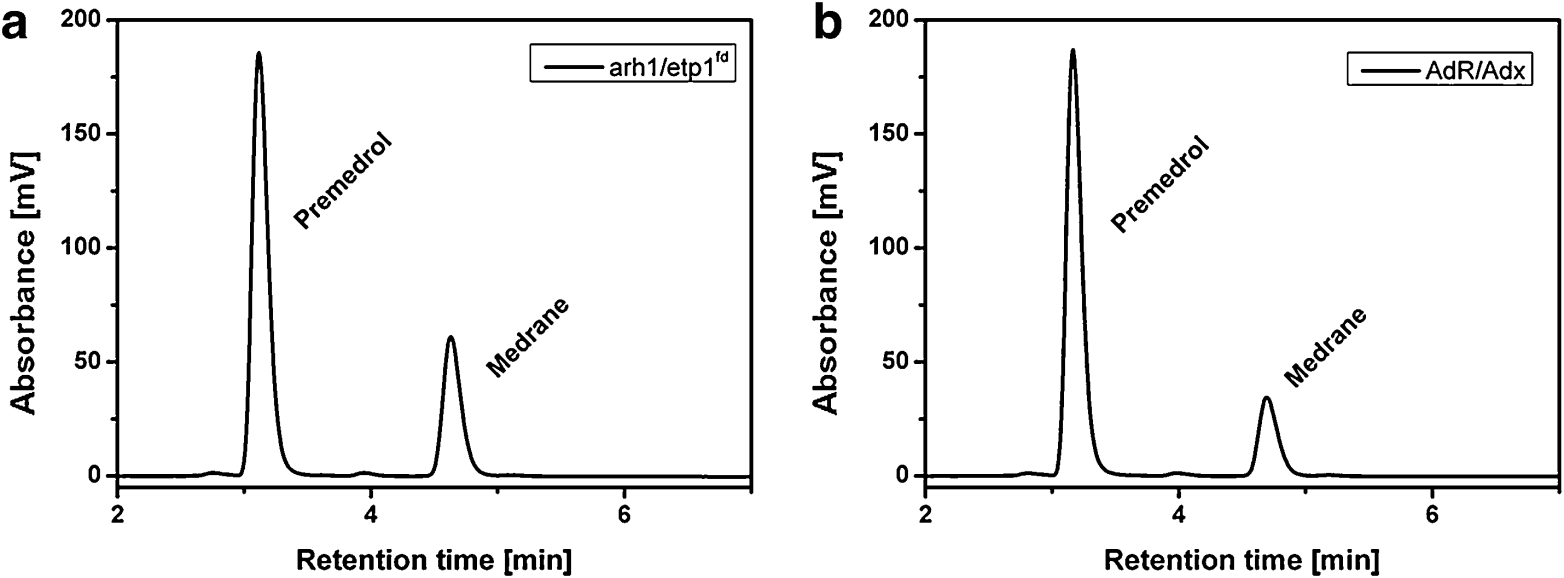
HPLC chromatograms of the in vitro biotransformation of medrane with bovine CYP21A2 and Adx or etp1^fd^ as final redox partner. Substrate conversions of medrane were performed with purified bovine CYP21A2 and **a** Adx as final electron donor, here with its natural reductase AdR, and **b** etp1^fd^ as final electron donor, here with its natural reductase arh1 for 30 min. Steroids were extracted and analyzed by HPLC to verify a selective conversion to premedrol, respectively

To verify whether Adx and etp1^fd^ as final electron transfer proteins are able to supply CYP21A2 with electrons, in vitro assays were carried out with different combinations of reductases and ferredoxins, listed in Table 1. HPLC analysis revealed, that both, Adx and etp1^fd^, are able to transfer electrons to CYP21A2, no matter which reductase was chosen (Fig. 4). It was shown for the first time, that etp1^fd^ is able to interact with CYP21A2 and, furthermore, to recruit the *E. coli* reductase Fpr as redox partner.

**Table 1:** Vectors for the *E. coli* whole-cell system

Vector	Reductase	Origin	Ferredoxin	Origin
p21b_bRED	CPR	*Bos taurus*		
p21b_AdAx	AdR	*Bos taurus*	Adx	*Bos taurus*
p21b_ArAx	arh1	*S. pombe*	Adx	*Bos taurus*
p21b_FrAx	Fpr	*E. coli*	Adx	*Bos taurus*
p21b_ArEt	arh1	*S. pombe*	etp1^fd^	*S. pombe*
p21b_FrEt	Fpr	*E. coli*	etp1^fd^	*S. pombe*

Six pET17b based vectors were constructed, each carrying the *CYP21A2* cDNA, in either a bicistronic construct with the cDNA for *CPR* or in a tricistronic construct with the cDNAs for *Adx*_1–108_ or *etp1* ^*fd*^ as final electron donors. The origin of the respective protein is mentioned

In the following, we show efforts to exchange the natural redox partner CPR by bovine Adx as well as etp1^fd^ as final electron donors in combination with different reductases in a whole-cell system to achieve an enhanced premedrol yield.

#### Construction of vectors for a whole-cell system with various redox chains

We constructed three vectors with a tricistronic transcription unit, each containing Adx as final electron donor. The vector p21b_ArAx was constructed containing the ORF for bovine CYP21A2, followed by the ORF for bovine AdR and Adx_1–108_, which represents the mitochondrial redox chain. Then, we replaced the AdR sequence with the one for arh1 from *S. pombe*. Finally, we used the *E. coli* reductase Fpr instead of AdR. The three resulting constructs are shown in Fig. S2 (Additional file 2).

Two more vectors were constructed, containing etp1^fd^ as electron donor of CYP21A2, on the one hand in combination with its natural ferredoxin reductase from *S. pombe,* arh1 (p21b_ArEt), on the other hand with the *E. coli* reductase, Fpr (p21b_FrEt). The resulting constructs are shown in Fig. S3 (Additional file 3). All constructed vectors are listed in Table 1.

#### Evaluation of the CYP21A2 whole-cell systems with different redox partners

**Fig. 5:**
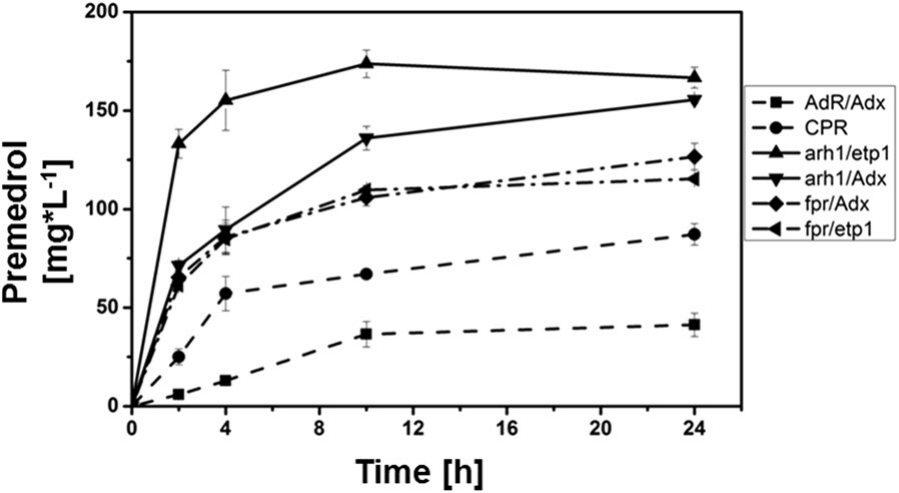
Time-dependent premedrol formation by different CYP21A2 based whole-cell systems. Whole-cell biotransformation was performed with all constructed systems simultaneously by using resting cells. Samples were taken after 0, 2, 4, 10 and 24 h and analyzed via HPLC. The premedrol yield at several time points was determined for each system. All values represent the mean of triplicates with the respective standard deviation

C43(DE3) cells were co-transformed with pGro12 and the constructed vectors, cultivated simultaneously to compare the initial productivity of each system as well as the endpoint yield of premedrol. Whole-cell biotransformation was carried out with resting cells in potassium phosphate buffer and samples for HPLC analysis were taken after 0, 2, 4, 6, 10 and 24 h to get a characteristic time course of the product formation depending on the respective redox system. A substrate concentration of 500 µM medrane was applied. HPLC analysis verified the biotransformation ability of each system. Regarding the endpoint yield, the systems containing the mitochondrial (AdR/Adx) and microsomal (CPR) redox partners produced the lowest amount of premedrol with 41 and 87 mg L^−1^ d^−1^, respectively. Taken the fact into account that AdR and CPR are membrane-associated and membrane-bound proteins, respectively, the recombinant synthesis and stability of these enzymes might be limited and, therefore, could represent a disadvantage for their application in a biotransformation process. Remarkably, the endpoint yield was dependent on the expressed reductases and not on the respective ferredoxin, suggesting that within this whole-cell system the functionality of the reductases is a limiting factor. The biotransformation overall yield with Fpr was higher than that with CPR with 127 and 115 mg L^−1^ d^−1^, no matter which ferredoxin, Adx or etp1^fd^, was the final electron donor for CYP21. The same was observed with arh1 as reductase with 156 mg L^−1^ d^−1^ together with Adx and 167 mg L^−1^ d^−1^ with its natural redox partner etp1^fd^, emphasizing that the soluble proteins, Fpr and arh1, are more suitable for a whole-cell system (Fig. 5; Table 2).

**Table 2:** Premedrol yield of different systems for whole-cell biotransformations performed with bCYP21A2 and various redox chains

Redox system	Overall yield premedrol (mg L^−1^ d^−1^)	Initial rate (mg L^−1^ h^−1^)
CPR	87 ± 5	13 ± 2
AdR/Adx	41 ± 6	3 ± 0.2
arh1/Adx	156 ± 2	36 ± 1
Fpr/Adx	127 ± 7	33 ± 2
arh1/etp1^fd^	167 ± 5	67 ± 4
Fpr/etp1^fd^	115 ± 1	31 ± 0.4

The premedrol overall yield was determined via HPLC analysis after 24 h conversion with all constructs simultaneously. The initial rate was calculated taking the first 2 h into account after starting the reaction by substrate addition. All values represent the mean of triplicates with the respective standard deviation

Though the endpoint yields of the different systems are similar when using the same reductase, the time course revealed a crucial difference regarding reaction velocity. While the velocities of the redox partner combinations arh1/Adx, Fpr/Adx and Fpr/etp1^fd^ are similar within the first 4 h, the system containing the reductase as well as the ferredoxin from *S. pombe* exhibits a higher efficiency with about doubled product formation between 2 and 4 h of substrate conversion, obviously due to the fact that arh1 together with etp1^fd^ represents a natural redox chain with optimal protein–protein interaction properties. Table 2 lists the initial and final product formation rates.

Taken together, we clearly demonstrated, that a redox protein exchange for the CYP21 whole-cell system increased the overall yield from about 90 mg L^−1^ d^−1^ to about 167 mg L^−1^ d^−1^, by use of arh1 and etp1^fd^ instead of CPR. It was also demonstrated that the reaction velocity is strongly dependent on the expressed redox proteins. With a substrate concentration of 1 mM medrane we could maximize the premedrol yield to 320 mg L^−1^ d^−1^ with the arh1 and etp1^fd^ redox chain in subsequent experiments. The fact that arh1 is able to receive electrons not only from NADPH but also from NADH (Additional file 3: Fig. S3) emphasizes the great potential of this reductase in a whole-cell application tapping an additional electron pool compared with the NADPH dependent Fpr and AdR, taking into account the fact that in *E. coli* NADH is the predominant co-factor under normal metabolic conditions of *E. coli* [44, 45].

To compare our established *E. coli* system with the human CYP21A2 based whole-cell system in *S. pombe*, we performed substrate conversions with the natural substrate 17OH-progesterone, since Zehentgruber et al. used it in an *S. pombe* whole-cell system [22]. With the *E. coli* system we achieved 308 ± 16 mg L^−1^ d^−1^ of the product 11-deoxycortisol, which is an about fourfold higher product yield compared with the system in *S. pombe* producing 77 mg product per L and day. Taken into account that Zehentgruber et al. used a cell density of 360 g L^−1^, which is tenfold higher than the applied *E. coli* density of 24 g L^−1^, we achieved a productivity of 37 µmol g^−1^ cell wet weight, while only 0.625 µmol g^−1^ cell wet weight were produced with the *S. pombe* system, which is 60 times less. This data clearly demonstrates the high efficiency and productivity of the established *E. coli* whole-cell system.

**Fig. 6:**
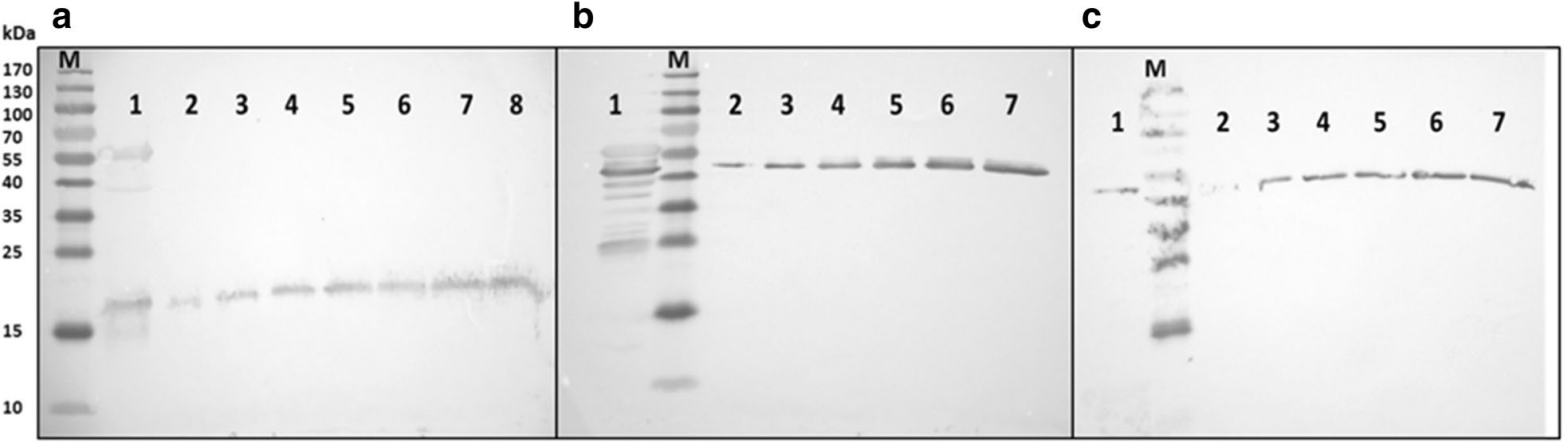
Western blot analysis of CYP21A2 and redox proteins in the whole-cell system for determination of the stoichiometric ratio of the three proteins encoded by the tricistronic construct p21b_ArEt. Western blot analysis was performed with polyclonal antibodies against **a** etp1^fd^ (11.3 kDa), **b** arh1 (51 kDa), c bovine CYP21A2 (54.6 kDa). In each blot, lane 1 represents the *E. coli* cell extract expressing arh1, etp1^fd^ and CYP21A2 after 28 h. **a** *lane 2–8* represents purified etp1^fd^ in increasing amounts (10, 20, 30, 40, 50, 75 and 100 ng), **b**, **c** The *lanes 2–7* show purified arh1 and CYP21A2 in increasing amounts (arh1: 25, 50, 75, 125, 187.5 and 250 ng, CYP21A2: 24, 48, 95, 143, 191 and 239 ng). The with “M” marked *lanes* all represent a prestained protein marker. The relative lane intensities, which correlate with the respective protein masses, were determined and compared to the intensity of the whole-cell system sample. Mass values were converted into the amount of substances and extrapolated to the expression yield per liter culture. Note that etp1^fd^ is known to give a single band in the range of the double mass expected to see on the SDS-PAGE

To examine the stoichiometry of CYP21A2:arh1:etp1^fd^, which are encoded by a tricistronic transcription unit, Western blot analysis was carried out for each enzyme after an expression time of 28 h according to Janocha et al. [41]. The highest expression level was determined for etp1^fd^ with approx. 880 nmol L^−1^. The reductase arh1 expression level is estimated to be approx. 498 nmol L^−1^ and the lowest one is for CYP21A2 with approx. 119 nmol L^−1^. Thus, the proteins are expressed with a ratio of 1:4:7 (CYP21A2:arh1:etp1^fd^) demonstrating an excess of reductase and ferredoxin, which supports a sufficient electron supply to CYP21A2 and underlines the system’s high efficiency (Fig. 6).

#### Determination of dissociation constants by difference spectroscopy

**Fig. 7:**
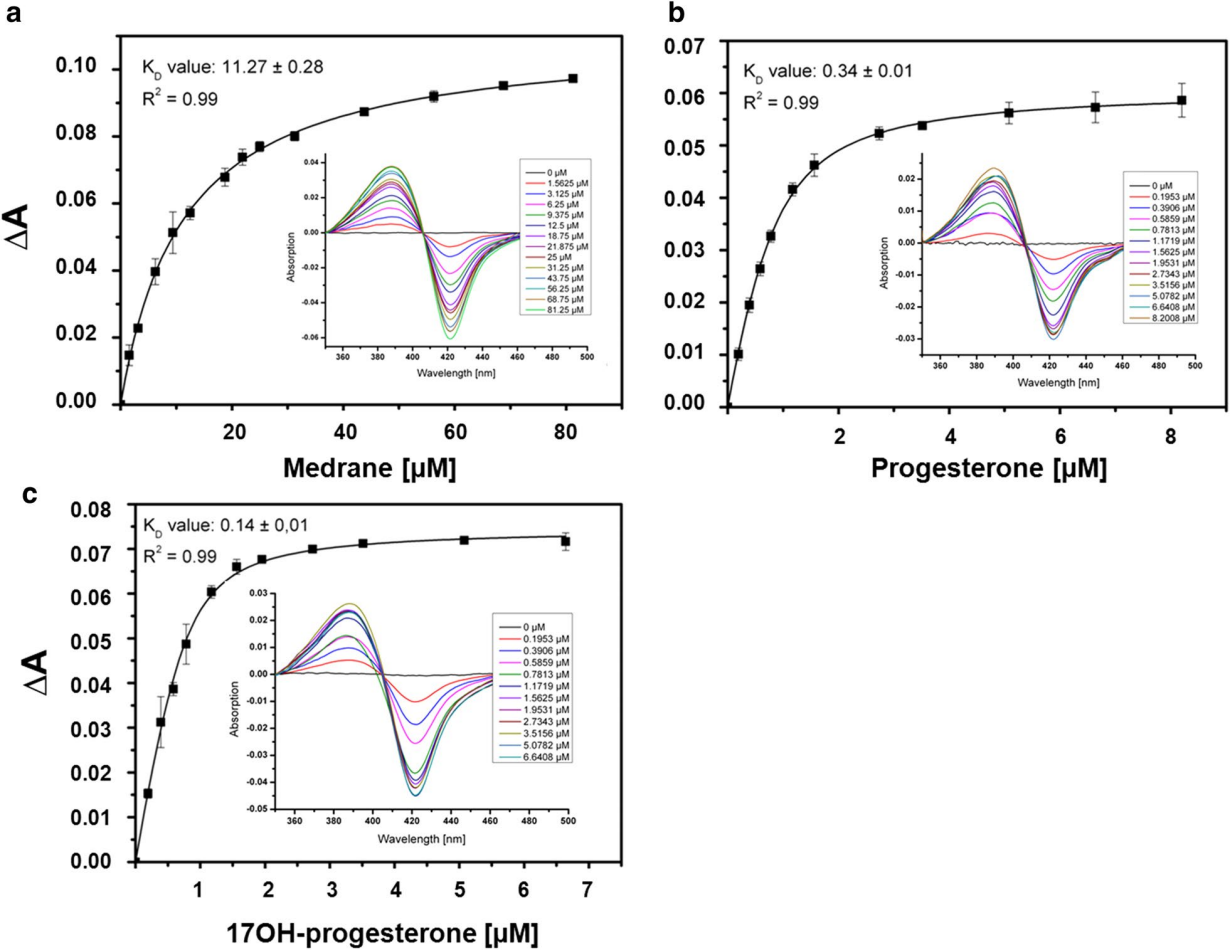
Determination of dissociation constants (K_D_ values) with different substrates titrated to CYP21A2. Dissociation constants for **a** medrane, **b** progesterone and **c** 17OH-progesterone were determined by titration of bovine CYP21A2 with increasing concentrations of the particular substrate leading to a typical type I shift. Hyperbolic regression resulted in the respective K_D_ values, indicating the extent of the binding affinity towards the enzyme

To compare the binding ability of bovine CYP21A2 to the synthetic substrate medrane to natural ones, progesterone and 17OH-progesterone, and to examine a possible limitation for medrane conversion due to a decreased protein binding, we determined the dissociation constant of the CYP21A2-medrane complex by difference spectroscopy. Complex formation between a potential substrate and CYP21A2 is spectroscopically detectable as type I shift due to the replacement of the heme coordinated H_2_O molecule. Titration of CYP21A2 with increasing amounts of medrane shows a typical type I shift (Fig. 7a) and, therefore, underlines a medrane conversion by CYP21A2. The difference of the absorbance maximum and minimum plotted against the substrate concentration of each titrating step results in a hyperbolic regression curve revealing a K_D_ value of 11.27 ± 0.28 µM for medrane. To compare the K_D_ value of the non-natural substrate medrane with those of the natural substrates, 17OH-progesterone and progesterone, we additionally titrated CYP21A2 with increasing amounts of these steroids. Hyperbolic regression resulted in a K_D_ value for 17OH-progesterone of 0.14 ± 0.01 µM and for progesterone of 0.34 ± 0.01 µM (Fig. 7b, c), implicating a higher affinity of natural CYP21A2 substrates compared with the synthetic one, which is due to the amino acid arrangement in the active site of CYP21A2 to ensure a selective 21-hydroxylation of the natural substrates [10]. Considering the nearly 100-fold higher K_D_ value of medrane compared with the natural substrate 17OH-P4, an enzyme improvement could aspire a stronger binding of the synthetic substrate to promote a more efficient premedrol production. This hypothesis is underlined by a higher product formation when using 17OH-progesterone as a substrate displaying a yield of 889 ± 59 µM d^−1^ 11-deoxycortisol compared with a premedrol yield of 640 ± 13 µM d^–1^. Hence, biotransformation with CYP21A2 using the natural substrate with the lowest dissociation constant shows a 40 % higher product formation than bioconversion with medrane.

### Scale-up of the whole-cell system with CYP21A2 and the redox partners arh1 and etp1^fd^

**Fig. 8:**
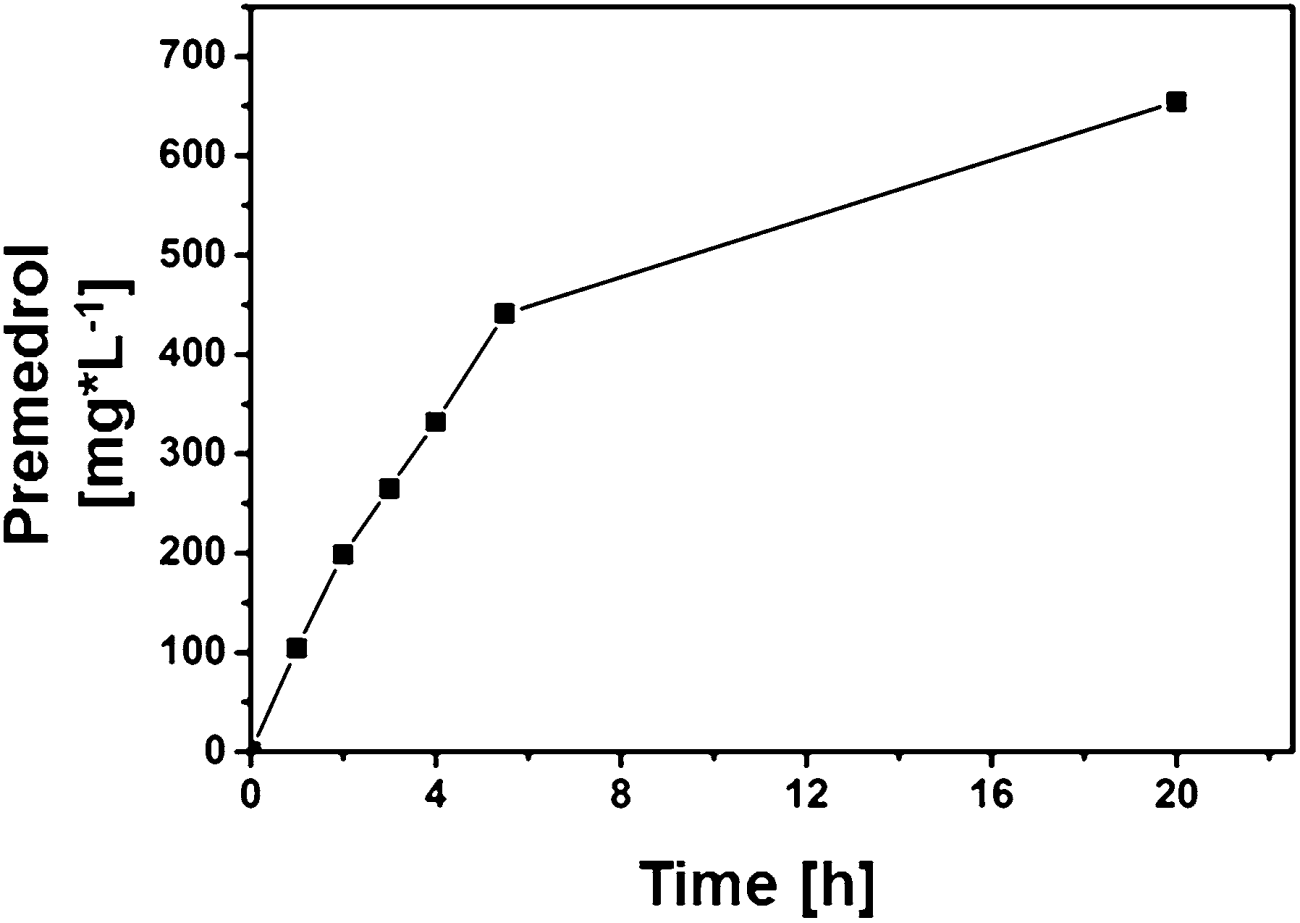
Time-dependent premedrol formation by the CYP21A2 based whole-cell system with arh1 and etp1^fd^ in a bioreactor. Whole-cell biotransformation in a bioreactor was performed with cells of C43(DE3), transformed with the vector p21b_ArEt, which is encoding bovine CYP21A2 as well as arh1 and etp1^fd^, by using resting cells in a volume of 500 mL and a cell density of 72 g L^−1^. Samples were taken after certain time points and analyzed via HPLC to examine the temporal premedrol increase

After the establishment of a CYP21A2 based whole-cell system and a further improvement of the system’s efficiency by alternative redox chains in shaking flasks, we pursued a scale-up of the system by a fermentation approach with increased cell density and the possibility to supplement oxygen by the stir velocity, since a sufficient oxygen supply is indispensable for CYP dependent reactions. Therefore, we performed a scale-up of the most efficient whole-cell system consisting of bovine CYP21A2 and the heterologous redox partner proteins arh1 and etp1^fd^. Protein expression was performed in Erlenmeyer flasks, and after a washing step the cell density for the biotransformation was adjusted to 72 g L^−1^. The reaction took place in the bioreactor BiostatQ^®^ with 500 mL resting cells in a defined buffer medium. 1000 mg medrane were added and the reaction was performed for 20 h with a stir velocity of 700 rpm. With this simple scale-up approach from a reaction volume of 25 mL in shaking flasks up to 500 mL in a bioreactor, a higher cell density was reached and a maximum product yield of 0.65 g premedrol per L d^−1^ could be achieved. Considering the reaction’s time dependence it was shown that the initial rate of 88 mg L^−1^ h^−1^ within the first 3 h dropped to 15 mg L^−1^ h^−1^ within the last 15 h of the biotransformation (Fig. 8). The decrease of the reaction rate was already observed in shaking flasks and in other CYP dependent whole-cell systems indicating limiting factors for a continuous biotransformation [46]. In case of CYP21A2, protein stability as a limiting factor could be excluded by CO difference spectroscopy of samples taken before and after bioconversion, which showed a highly stable enzyme (Additional file 4: Fig. S4). Furthermore, we could confirm by Western blot analysis that there exists an optimal stoichiometry of CYP21A2 and the redox proteins arh1 and etp1^fd^. Regarding a biotechnological application the next step would be the establishment of a controlled fermentation process ensuring a stable pH, carbon source as well as substrate feeding and, overall, a sufficient supply with oxygen needed for a CYP catalyzed reaction. Nevertheless, this scale-up approach implicates the potential to increase the whole-cell system’s efficiency by a biotransformation in a bioreactor and already produced nearly gram amounts of product per liter and day (Additional file 5: Fig. S5, Additional file 6: Table S1).

## Conclusions

In this work, we demonstrated that the mammalian CYP21A2, which is involved in mammalian steroid hormone biosynthesis and catalyzes a stereo- and regioselective 21-hydroxylation of progesterone and 17OH-progesterone, also exhibits a high potential as biocatalyst for medrol production. It could be demonstrated with purified enzyme as well as in a newly established *E. coli* whole-cell system that CYP21A2 is able to convert medrane to premedrol, an important precursor of medrol, via a selective oxofunctionalization at C21. Since a sufficient electron supply is an essential factor influencing CYP dependent reactions, the focus has shifted from the natural CYP21A2 redox partner, the membrane protein CPR, to alternative redox proteins. Therefore, five different redox systems were introduced and examined regarding velocity and endpoint yield. The systems containing the ferredoxin reductase arh1 from *S. pombe* were similar in their endpoint independent on the used ferredoxin. With regard of the initial reaction rate it is shown in Fig. 5, that the redox chain consisting of arh1 and its natural ferredoxin etp1^fd^ is two times faster than all other systems, possibly due to the fact that the electron transfer between a natural redox pair is much more effective and faster than in the system with Adx (Fig. 5; Table 2). Taken together, the use of soluble enzymes with a high expression yield seems to be the best choice for the system’s stability and effectiveness, which is underlined by an ideal protein stoichiometry, confirmed by Western blot analysis of the three participating proteins (Fig. 6). Compared to the CPR based system the premedrol yield could be enhanced about the 3.6 fold by the use of other redox partners, which shows the high impact of a sufficient, stable and suitable electron supply during biotransformation. We could achieve 320 mg L^−1^ d^−1^ in shaking flasks experiments. A scale-up approach to 500 mL in a bioreactor, including an increase of the cell density, could further improve the overall yield up to a maximum of 0.65 g L^−1^ d^−1^ which shows the potential of the system for an industrial application and an important step towards the replacement of the chemical premedrol synthesis by a biocatalytic approach.

## Methods

### Chemicals, kits, enzymes and primary antibodies

All chemicals were from standard sources and of highest purity. Solvents used for chromatographic analysis were of gradient grade. Steroids for analysis and whole-cell biotransformation were from Sanofi, Frankfurt-Höchst (DE) and of highest purity. Restriction enzymes were obtained from New England Biolabs (Frankfurt, DE), kits for plasmid preparation and DNA purification from Machery-Nagel (Düren, DE) and the FastLink™ DNA Ligation Kit from Epicentre Biotechnologies (Madison, US). The primary antibody against arh1 was obtained from Charles River Laboratories (Sulzfeld, Germany), against etp1^fd^ from BioGenes (Berlin, Germany) and against bovine CYP21A2 from antikoerper-online.de (Aachen, Germany).

### Bacterial strains and cultivation

Plasmid preparation and cloning experiments were carried out with *E. coli* TOP10 (Invitrogen, San Diego, CA, USA). Protein synthesis and whole-cell biotransformation were performed with *E. coli* strain C43(DE3) [47]. The cultivation took place in Luria–Bertani broth (BD, Heidelberg, DE) or in terrific broth (TB) complex medium. Transformed cells were stored as glycerol stock with a 1:1 mixture of an overnight culture and glycerol (50 %) at −80 °C.

### Molecular cloning

#### Expression vector for bovine and human CYP21A2

The cDNAs for bovine and human *CYP21A2* was constructed according to Arase et al. with a replacement of the N-terminal hydrophobic anchor region with MAKKTSSKGK from CYP2C3 and a 6× Histidin tag for protein purification [24, 48]. It was digested with NdeI and BamHI and ligated into the pET17b expression vector (Novagen). The constructed vectors are subsequently designated as pET17b_21b and pET17b_21h.

#### Construction of vectors for a whole-cell biotransformation

All constructed vectors for a whole-cell biotransformation are based on the pET17b expression vector. All constructs consist of bi- or tricistronic transcription units with the *CYP21A2* cDNA sequence and one or two redox partner cDNAs downstream of it (Fig. 2, Additional file 1: Fig S1, Additional file 2: S2).

The vector pET17b_21b served as a backbone for the construction of the vector p21b_bRED, containing the cDNA for bovine *CYP21A2* and its natural redox partner, the bovine cytochrome P450 reductase (*CPR*), as a bicistronic transcription unit. A CPR containing vector was used for PCR amplification of *CPR* cDNA [49]. The forward primer contains a BamHI restriction side followed by a ribosomal binding side and the respective coding region. The appropriate reverse primer carries the C-terminal coding region including the stop codon and a NotI restriction side. The PCR product was digested and ligated between the BamHI and NotI sides of pET17b_21b, resulting in the bicistronic construct p21b_bRED. The vector p21h_bRED was cloned likewise.

The vector p21b_AdAx contains a tricistronic transcription unit, consisting of the cDNA for bovine *CYP21A2*, bovine adrenodoxin reductase (*AdR*) and truncated bovine adrenodoxin (*Adx*_*1*–*108*_). Bovine AdR and Adx represent the mitochondrial redox system, which is proved to interact with CYP21A2. The vector Twin11B1 served as a backbone for the construction and carries the cDNA for human *CYP11B1*, bovine *AdR* and bovine *Adx* in a tricistronic arrangement [20]. Firstly, an undesired HindIII site had to be removed within the *CYP21A2* sequence by QuikChange^®^ Site-Directed Mutagenesis. The resulting cDNA for bovine *CYP21A2* was amplified by PCR with pET17b_21b as a template. The forward primer is equal to the existing DNA sequence and contains an NdeI restriction site. The reverse primer carries the end of the coding region and a HindIII site. Both PCR product and the vector Twin11B1 were digested and the *CYP21A2* cDNA was ligated between the NdeI and HindIII sites of Twin 11B1 by a replacement of the *CYP11B1* sequence against the *CYP21A2* cDNA which results in the tricistronic vector p21b_AdAx.

The vector p21b_FrAx was constructed according to p21b_AdAx and contains the *E. coli* reductase Fpr instead of AdR, which is cloned through the HindIII and KpnI sites. Origin of the Fpr sequence was the vector pET_MR6 [27].

For cloning of the vectors harboring components of the redox system from *S. pombe* the vector pBar_Twin_pombe served as a template, carrying the cDNA for adrenodoxin reductase homologue 1 (*arh1*) and the ferredoxin domain of the electron transfer protein 1 (*etp1*^*fd*^) [36, 38, 41]. In a first step, the AdR sequence of the vector p21b_AdAx was replaced by the *arh1* cDNA which was amplified via PCR using pBar_Twin_pombe as a template. The forward primer carried a HindIII restriction site as well as a following ribosomal binding site while the reverse primer was identical to the C-terminal sequence including a KpnI restriction site. The amplified PCR product was digested and cloned into the likewise digested p21b_AdAx. The resulting vector p21b_ArAx contains a tricistronic construct composed of the cDNAs for *CYP21A2*, *arh1* and *Adx.*

In the next step, the vector p21b_ArEt was constructed based on the backbone of the vector p21b_ArAx, which contains both components of the *S. pombe* redox system. Again, pBar_Twin_pombe served as a template for a PCR amplification of *etp1*^*fd*^. The forward as well as the reverse primer were identical to the etp1^fd^ cDNA sequence, carrying a KpnI and an EcoRI restriction site. The PCR product was digested and ligated between the KpnI and EcoRI restriction sites of the likewise digested p21b_ArAx resulting in an exchange of Adx by etp1^fd^. The vector p21b_FrEt was cloned likewise. All used primers are listed in Additional file 6: Table S6.

### Protein expression and purification

#### Expression and purification of electron transfer proteins

Bovine AdR and Adx as well as arh1, Fpr and etp1^fd^ were expressed in *E. coli* and purified as described before [27, 34, 36, 38, 50].

Bovine CPR was synthesized in *E. coli* and purified via Immobilized Metal Ion Affinity Chromatography (IMAC) as described elsewhere [49].

### Expression and purification of bovine and human CYP21A2

C43(DE3) were co-transformed with the expression vector pET17b_21b and the vector pGro12 which carries the genes for the molecular *E. coli* chaperones GroEL/ES to ensure a proper protein folding and a correct integration of the heme cofactor. For the seed culture, 10 mL LB medium, supplemented with 100 µg mL^−1^ ampicillin for pET17b_21b selection and 50 µg mL^−1^ kanamycin for pGro12 selection, were inoculated with transformed cells from a glycerol stock and grown overnight at 37 °C with 160 rpm. For the main culture, 250 mL TB medium, supplemented with 100 µg mL^−1^ ampicillin and 50 µg mL^−1^ kanamycin, were inoculated with 1/100 (v/v) of the seed culture and grown at 37 °C with 190 rpm to an OD_600_ of 0.5. At this time point, gene expression was induced by adding 1 mM isopropylthiogalactopyranosid (IPTG), 1 mM δ-aminolevulinic acid as heme precursor and 4 mg mL^−1^l-arabinose for the induction of the chaperones GroEL/ES. Protein synthesis was carried out at 27 °C with 150 rpm for 38 h. Cells were harvested at 4,000*g* for 20 min at 4 °C.

Cell pellets were diluted in lysis buffer, consisting of 50 mM potassium phosphate buffer (pH 7.4), 500 mM sodium acetate, 0.1 mM EDTA, 20 % glycerin, 1.5 % sodium cholate, 1.5 % Tween20, 0.1 mM PMSF and 0.1 mM DTE. Cells were disrupted by sonification and centrifuged with 30,000*g* at 4 °C for 30 min. The supernatant was taken for the subsequent purification. The 3 step protein purification by Immobilized Metal Ion Affinity Chromatography (IMAC) and DEAE Sepharose as well as SP Sepharose for ion exchange chromatography was done as previously described by Arase et al. [24].

### UV/vis spectroscopy

CO difference spectroscopy of reduced CYP in complex with CO was carried out for a qualitative and quantitative enzyme characterization following the typical absorption maximum at 450 nm with an extinction coefficient of 91 mM^−1^ cm^−1^ [13].

Difference spectroscopy was performed to examine the binding behavior of CYP21A2 natural and unnatural CYP21A2 substrates as previously described by using tandem cuvettes. CYP21A2 was dissolved in buffer (50 mM potassium phosphate (pH 7.4), 20 % glycerol, 0.5 % sodium cholate and 0.05 % Tween 20) and titrated with increasing amounts of substrate in DMSO. Difference spectra were recorded from 350 to 500 nm. The values from three titrations were averaged and the K_D_ values were determined by fitting the plots with hyperbolic regression or tight binding quadratic equation with OriginPro 9.1G [51].

### Reconstituted in vitro assays

The in vitro reconstitution assay was performed in a final volume of 250 µL with 50 mM HEPES buffer (pH 7.4) containing either 100 µM DLPC and 20 % glycerol for the CPR or 0.5 % Tween20 for all other redox proteins. The final concentration of CYP21A2 was 0.5 µM, the concentration of arh1 and AdR 0.5 µM, of Adx and etp1^fd^ 10 µM, of the Fpr 25 µM and of the CPR 1 µM, respectively. Additionally, the mixture contained a NADPH regeneration system consisting of 5 mM glucose-6-phosphate, 1 mM MgCl_2_ as well as glucose-6-phosphate dehydrogenase. The particular steroid substrate was added in a concentration range of 100–400 µM. The reaction was started with 5 mM NADPH or NADH and incubated shaking for 30–40 min at 37 °C. The assay was stopped by addition of 250 µL chloroform, steroids were extracted twice with chloroform, dried and stored at −20 °C for HPLC analysis.

### Whole-cell biotransformation with different redox systems in shaking flasks

Protein synthesis of bovine and human CYP21A2 and the respective redox partners for a whole-cell biotransformation was performed as described above by co-transformation of C43(DE3) cells with the particular bi-or tricistronic vector and the pGro12. After 28 h expression time, cells were harvested at 4,000*g* for 15 min at room temperature. The cell pellets were washed with 50 mM potassium phosphate buffer and cell wet weight was adjusted to 24 g L^−1^. The whole-cell biotransformation was carried out with resting cells in 50 mM potassium phosphate buffer (pH 7.4) supplemented with 2 % glycerol, 1 mM IPTG, 1 mM δ-Ala, 4 mg mL^−1^ arabinose and 25 µg mL^−1^ polymyxin B. The reaction volume was 25 mL in 300 mL baffled Erlenmeyer flasks. The steroid substrate was solved in DMSO and added in concentrations ranging from 200 to 1.2 mM. The whole-cell reaction mixture was incubated at 27 °C with 145 rpm for 24 h. Samples for HPLC analysis were taken at different time points, extracted twice with chloroform, dried and stored at −20 °C.

### Whole-cell biotransformation in a bioreactor

Protein synthesis of bovine CYP21A2 and the redox partners arh1 and etp1^fd^ for a whole-cell biotransformation was performed as described above, by co-transformation of C43(DE3) cells with the p21b_ArEt vector and the pGro12. After 28 h expression time, cells were harvested at 4,000*g* for 15 min at room temperature. The cell pellets were washed with 50 mM potassium phosphate buffer and cell wet weight was adjusted to 72 g L^−1^. The whole-cell biotransformation was carried out with resting cells in 50 mM potassium phosphate buffer (pH 7.4) supplemented with 2 % glycerol, 1 mM IPTG, 1 mM δ-Ala, 4 mg mL^−1^ arabinose and 25 µg mL^−1^ polymyxin B. The reaction volume was 500 mL and the biotransformation was carried out in the bioreactor BiostatQ^®^ with a stir velocity of 700 rpm at 27 °C. The steroid substrate was dissolved in DMSO and added in concentrations up to 1000 mg L^−1^.

### Steroid analysis via RP-HPLC

Steroid analysis was carried out by reversed-phase high performance liquid chromatography using a Jasco reversed phase HPLC system of the LC900 series and a 4.6 mm × 125 mm NucleoDur C18 Isis Reversed Phase column (Macherey–Nagel).

The reconstituted in vitro assays were analyzed with an acetonitril/water gradient at 240 nm within 15 min at 40 °C and a flow rate of 0.8 mL min^−1^.

The whole-cell conversion was measured with an acetonitril/water gradient at 240 nm within 30 min at 40 °C and a flow rate of 0.8 mL min^−1^.

### Western blot analysis

Samples from the culture, co-expressing bovine CYP21A2, arh1 and etp1^fd^, were taken, adjusted to OD 1 and centrifuged. The pellet was suspended in 100 µl SDS-PAGE loading buffer and boiled for 10 min. 6 µL of the sample in case of CYP21A2 and arh1 and 3 µL in case of etp1^fd^ were separated on a 12 % acrylamide gel according to Laemmli et al. [52]. For Western blot analysis, proteins were transferred to hybond-ECL nitrocellulose membranes (Amersham, GE Healthcare, England) [53]. The membranes were blocked overnight in 3 % milk powder in 30 mL TBS (50 mM Tris–Cl pH 7.5, 400 mM NaCl, 0.15 % Tween 20). After blocking, the membranes were washed three times for 15 min with TBS and afterwards incubated for 1.5 h with the respective primary antibody, dissolved 1:1000 in TBS. After three following washing steps with TBS, incubation with the secondary horseradish-linked goat antirabbit IgG antibody (Dako, Glostrup, Denmark), diluted 1:3000 in TBS, took place for 1.5 h. In the following step, the membranes were washed three times for 15 min with PBS (10 mM potassium phosphate buffer pH 7.4, 150 mM NaCl) and afterwards, the protein-antibody conjugates were visualized by addition of 4-chloro-1-naphthol (2 mL; 3 mg/mL in ethanol) in 25 mL PBS supplemented with 10 μL H_2_O_2_. Relative intensity of the protein bands was measured with Image Lab 3.0 from BioRad (München, Germany). The determination of the protein yield was performed by comparing the sample amount (Fig. 6a–c, lane 1) to increasing concentrations of purified protein, for etp1^fd^ 10, 20, 30, 40, 50, 75 and 100 ng (Fig. 6a, lanes 2–8), for arh1 25, 50, 75, 125, 187.5 and 250 ng (Fig. 6b, lanes 2–7) and for bovine CYP21A2 24, 48, 95, 143, 191 and 239 ng (Fig. 6c, lanes 2–7). The relative lane intensities, which correlate with the respective protein masses, were determined and compared to the intensity of the whole-cell system sample. Mass values were converted into the amount of substances and extrapolated to the expression yield per liter culture.

## Additional files

**Additional file 1: Fig. S1.** HPLC chromatograms of the *in vivo* and *in vit*ro conversion of medrane with human CYP21A2 and CPR. **a** Medrane was converted with resting cells of C43(DE3) containing the human CYP21A2 isoform and CPR encoding vector p21h_bRED. Samples were taken after 24 h and extracted for HPLC analysis. The steroids were separated by an acetonitril:water gradient. **b** Substrate conversions of medrane were performed with purified human CYP21A2 and its redox partner CPR for 30 min. Steroids were extracted and analyzed by HPLC to verify a selective conversion of medrane to premedrol, respectively. The steroids were separated by an acetonitril:water gradient, showing the 21-hydroxylated product premedrol.
**Additional file 2: Fig. S2.** Vector maps of constructed vectors for a CYP21A2 based whole-cell system in *E. coli* with Adx as final electron donor. Three vectors with tricistronic transcription units were constructed, based on the pET17b vector with an inducible T7 promoter and an ampicillin resistance gene, with Adx as final electron donor in combination with one of the reductases, AdR (p21b_AdAx), arh1 (p21b_ArAx) or Fpr (p21b_FrAx).
**Additional file 3: Fig. S3.** Vector maps of constructed vectors for a CYP21A2 based whole-cell system in *E. coli* with etp1^fd^ as final electron donor. Two vectors with tricistronic transcription units were constructed, based on the pET17b vector with an inducible T7 promotor and an ampicillin resistance gene, with etp1^fd^ as final electron donor in combination with the reductases arh1 (p21b_ArEt) or Fpr (p21b_FrEt).
**Additional file 4: Fig. S4.** *In vitro* conversion of medrane with the redox systems AdR/Adx/CYP21A2 or arh1/Adx/CYP21A2 with either NADH or NADPH. 400 μM Medrane was converted in a reconstituted *in vitro* assay with Adx based redox systems containing AdR or arh1 as reductase. To each system either NADH or NADPH was added to verify the ability of arh1 to receive electrons from NADH. AdR served as a negative control. All values represent the mean of triplicates with the respective standard deviation.
**Additional file 5: Fig. S5.** CO difference spectra of bovine CYP21A2 before and after 24 h biotransformation. Cells were harvested before and after 24 hours biotransformation. COD was performed with the respective lysate, each showing a typical absorption maximum at 450 nm in a reduced state in complex with CO which indicates a correct heme insertion. The solid line shows the COD before biotransformation, the dashed one after 24 h bioconversion in buffer.
**Additional file 6: Table S1.** Sequences of used primers with indication of their purpose. Restriction sites are in bold letters and base exchanges are signed in bold and cursive.

## Abbreviations

CYP21A2: 21-hydroxylase
*E. coli*: *Escherichia coli*
