# Supplementary figures and images for: A CYP21A2 based whole-cell system in Escherichia coli for the biotechnological production of premedrol
Source: Microb Cell Fact. 2015 Sep 15;14:135. doi: 10.1186/s12934-015-0333-2 (PMC4572648; doi:10.1186/s12934-015-0333-2)

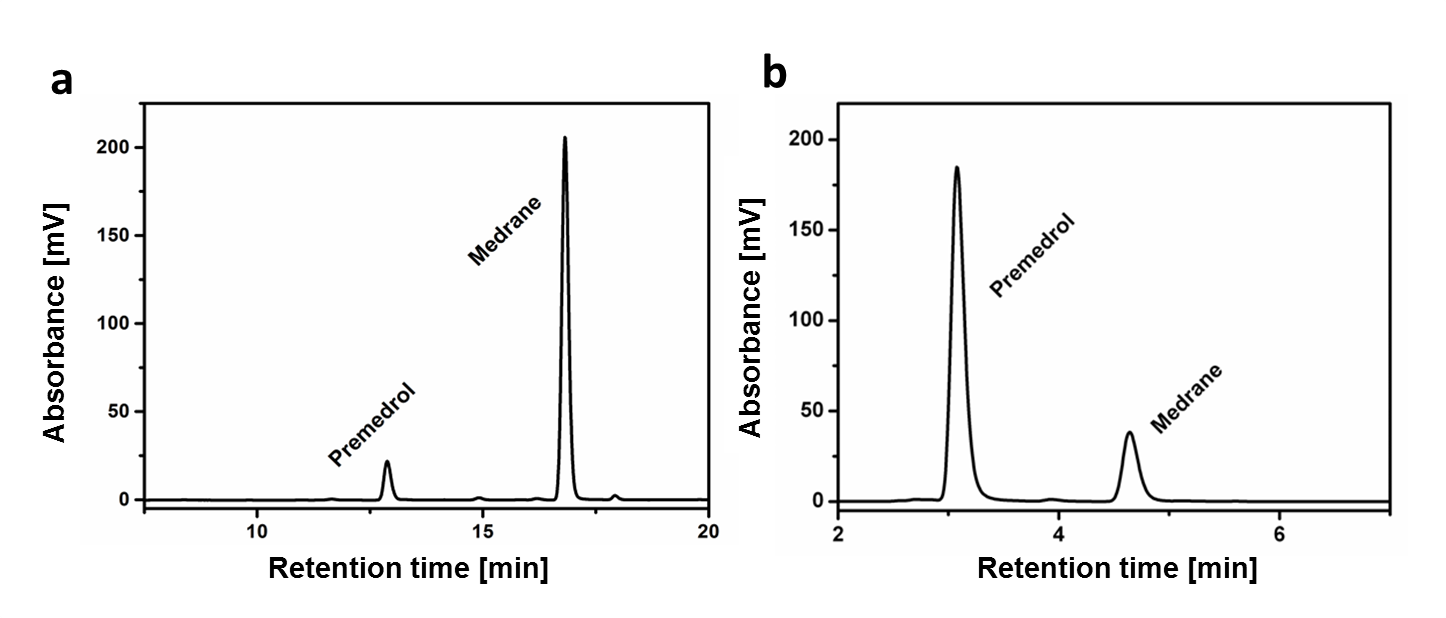

Supplement: Supplementary file 1 — Additional file 1: Fig. S1. HPLC chromatograms of the in vivo and in vitro conversion of medrane with human CYP21A2 and CPR. a Medrane was converted with resting cells of C43(DE3) containing the human CYP21A2 isoform and CPR encoding vector p21h_bRED. Samples were taken after 24 h and extracted for HPLC analysis. The steroids were separated by an acetonitril:water gradient. b Substrate conversions of medrane were performed with purified human CYP21A2 and its redox partner CPR for 30 min. Steroids were extracted and analyzed by HPLC to verify a selective conversion of medrane to premedrol, respectively. The steroids were separated by an acetonitril:water gradient, showing the 21-hydroxylated product premedrol. [file 12934_2015_333_MOESM1_ESM.tif]

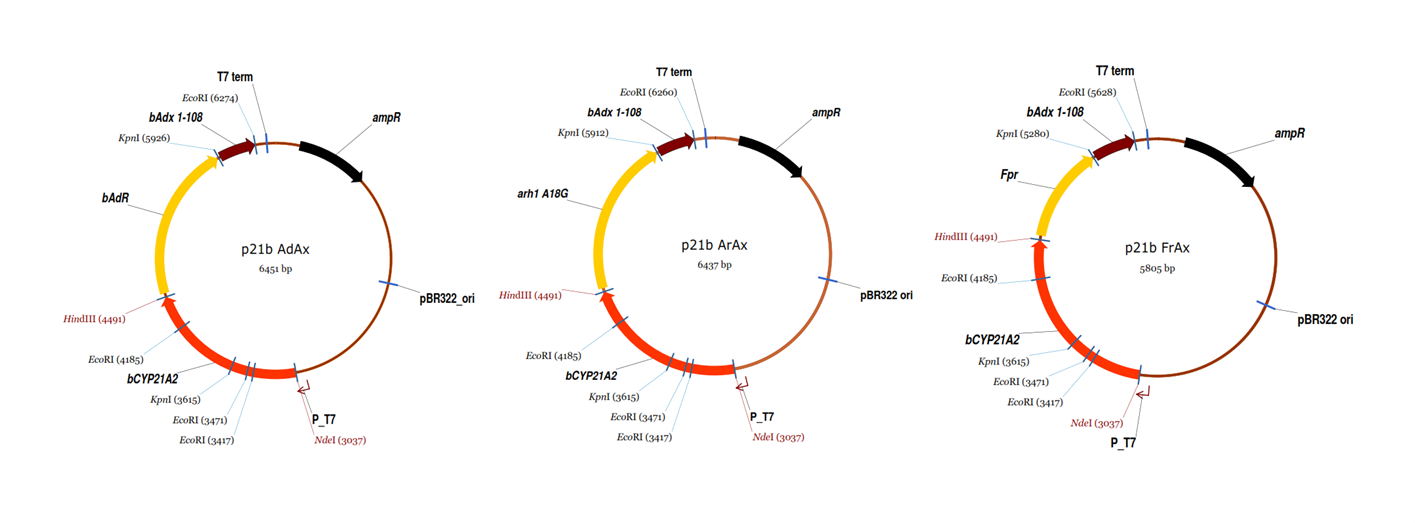

Supplement: Supplementary file 2 — Additional file 2: Fig. S2. Vector maps of constructed vectors for a CYP21A2 based whole-cell system in E. coli with Adx as final electron donor. Three vectors with tricistronic transcription units were constructed, based on the pET17b vector with an inducible T7 promoter and an ampicillin resistance gene, with Adx as final electron donor in combination with one of the reductases, AdR (p21b_AdAx), arh1 (p21b_ArAx) or Fpr (p21b_FrAx). [file 12934_2015_333_MOESM2_ESM.tif]

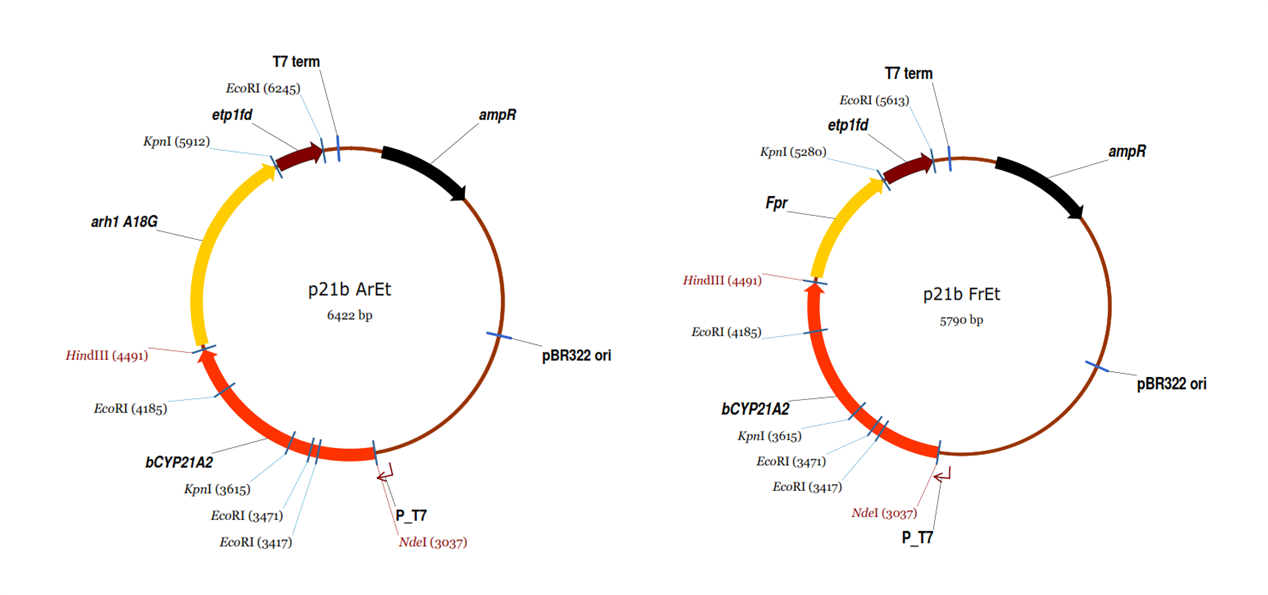

Supplement: Supplementary file 3 — Additional file 3: Fig. S3. Vector maps of constructed vectors for a CYP21A2 based whole-cell system in E. coli with etp1fd as final electron donor. Two vectors with tricistronic transcription units were constructed, based on the pET17b vector with an inducible T7 promotor and an ampicillin resistance gene, with etp1fd as final electron donor in combination with the reductases arh1 (p21b_ArEt) or Fpr (p21b_FrEt). [file 12934_2015_333_MOESM3_ESM.tif]

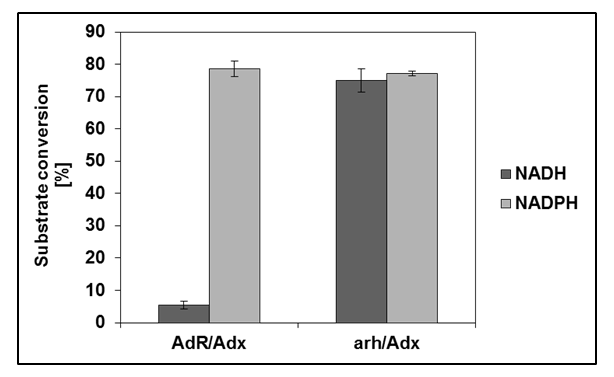

Supplement: Supplementary file 4 — Additional file 4: Fig. S4. In vitro conversion of medrane with the redox systems AdR/Adx/CYP21A2 or arh1/Adx/CYP21A2 with either NADH or NADPH. 400 μM Medrane was converted in a reconstituted in vitro assay with Adx based redox systems containing AdR or arh1 as reductase. To each system either NADH or NADPH was added to verify the ability of arh1 to receive electrons from NADH. AdR served as a negative control. All values represent the mean of triplicates with the respective standard deviation. [file 12934_2015_333_MOESM4_ESM.tif]

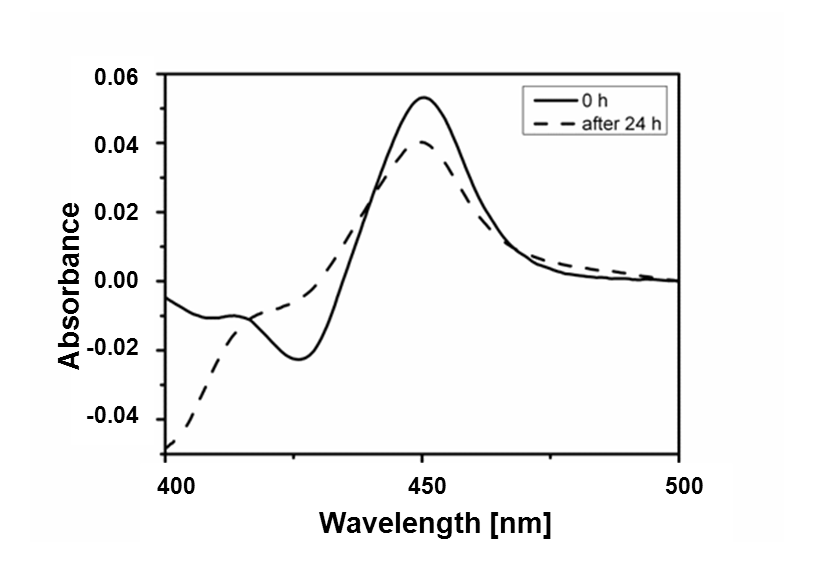

Supplement: Supplementary file 5 — Additional file 5: Fig. S5. CO difference spectra of bovine CYP21A2 before and after 24 h biotransformation. Cells were harvested before and after 24 hours biotransformation. COD was performed with the respective lysate, each showing a typical absorption maximum at 450 nm in a reduced state in complex with CO which indicates a correct heme insertion. The solid line shows the COD before biotransformation, the dashed one after 24 h bioconversion in buffer. [file 12934_2015_333_MOESM5_ESM.tif]
