# Supplementary material for: A CYP21A2 based whole-cell system in Escherichia coli for the biotechnological production of premedrol
Source: Microb Cell Fact. 2015 Sep 15;14:135. doi: 10.1186/s12934-015-0333-2 (PMC4572648; doi:10.1186/s12934-015-0333-2)
Supplement: Supplementary file 6 — Additional file 6: Table S1. Sequences of used primers with indication of their purpose. Restriction sites are in bold letters and base exchanges are signed in bold and cursive. [file 12934_2015_333_MOESM6_ESM.docx]

**Supplementary material**

**Table S1: Sequences of used primers with indication of their purpose.** Restriction sites are in bold letters and base exchanges are signed in bold and cursive.

| **Primer** | **Sequence 5´ 3´** | **Application** |
| --- | --- | --- |
| F_Cpr_Bam | **GGATCC**TTGTTTAACTTTAAGAAGGAGATATACATAT***G***AACATGGC | Amplification of *CPR* cDNA for construction of vector p21b_bRED |
| R_Cpr_Not | **GCGGCCGC**TCAGTGGTGGTGGTGGTGGTGAGA | Amplification of *CPR* cDNA for construction of vector p21b_bRED |
| F_bCYP21_delHindIII | GGTTATTACA***G***GCTTTTACATTATTACCACCCC | QC mutagenesis for removal of HindIII side in the *CYP21A2* cDNA |
| R_bCYP21_delHindIII | GTAATAATGTAAAAGC***C***TGTAATAACCTGAGGAG | QC mutagenesis for removal of HindIII side in the *CYP21A2* cDNA |
| F_CYP21_NdeI | GGAGATATA**CATATG**GCTAAAAAAACATCTTC | Amplification of *CYP21A2* cDNA for construction of vectors p21b_AdAx and p21b_FrAx |
| R_CYP21_HindIII | CATG**AAGCTT**TTAGTGGTGGTGGTGGTGGTG | Amplification of *CYP21A2* cDNA for construction of vectors p21b_AdAx and p21b_FrAx |
| F_arh_HindIII | CATG**AAGCTT**TTGTTTAACTTTAAGAAGGAGATATACCATGTCTACTCAAACTTCTTCACC | Amplification of *arh1* cDNA for construction of vector p21b_ArAx |
| R_arh_KpnI | CATG**GGTACC**TTATTTTTTACCGGGATATATTAGTTTAATC | Amplification of *arh1* cDNA for construction of vector p21b_ArAx |
| F_etp1 | CATGGGTACCAAGAAGGAGATATACCATGG | Amplification of e*tp1^fd^* cDNA for construction of vector p21b_ArEt and p21b_FrEt |
| R_etp1 | CATGGAATTCCCGGGTTAAGGGATTC | Amplification of *etp1^fd^* cDNA for construction of vector p21b_ArEt and p21b_FrEt |
